# Artificial liver support with Cytosorb and continuous veno-venous hemodiafiltration versus advanced organ support (ADVOS) for critically ill patients with hyperbilirubinemia and acute-on-chronic liver failure (ACLF)

**DOI:** 10.1186/s12882-025-04342-6

**Published:** 2025-08-04

**Authors:** Kristina Schönfelder, Luisa Katharina Hirsch, Andreas Kribben, Michael Jahn, Bartosz Tyczynski, Justa Friebus-Kardash

**Affiliations:** 1 Department of Nephrology, University of Duisburg-Essen, University Hospital Essen, Essen Hufelandstr. 55, 45147 Essen, Germany; 2 Department of Cardiology, Electrophysiology, Nephrology, Geriatric Medicine and Intensive Care Medicine, Alfred Krupp Hospital, Essen, Germany; 3 Department of Intensive Care Medicine I, University Hospital Essen, Essen, Germany

**Keywords:** CytoSorb, ADVOS, Bilirubin, Acute-on-chronic liver failure, Secondary acquired liver dysfunction, Continuous veno-venous hemodiafiltration

## Abstract

**Background:**

As many as 30% of critically ill patients in intensive care units experience acute liver dysfunction with hyperbilirubinemia as a part of multiorgan failure that is associated with poor outcome. This retrospective cohort study was aimed at comparing CytoSorb and ADVOS in terms of bilirubin removal and overall survival among critically ill patients with hyperbilirubinemia ≥ 7 mg/dL.

**Methods:**

At the University Hospital Essen, between January 2021 and March 2024, 71 patients were treated with CytoSorb integrated in a continuous veno-venous hemodiafiltration (CVVHDF) circuit, and 71 patients were treated with ADVOS. Each therapy session lasted 24 h. We separately analyzed the subgroup of patients with acute-on-chronic liver failure (ACLF), in which 31 patients were treated with CytoSorb and 66 patients were treated with ADVOS.

**Results:**

The first single sessions of both CytoSorb with CVVHDF and ADVOS were associated with a statistically significant decrease in total serum bilirubin levels (Cytosorb, 20 to 14 mg/dL, *p* < 0.0001; ADVOS, 16 to 14 mg/dL, *p* < 0.0001), but the percentage bilirubin reduction was more pronounced for CytoSorb treatment (26% vs. 17%, *p* = 0.0002). The number of days of treatment was similar for both groups (3 vs. 4, *p* = 0.07). After completion of therapy, serum levels of total bilirubin had decreased significantly; 19.9 to 11.3 mg/dl (*p* < 0.0001) in the CytoSorb group and 16.3 to 14.0 mg/dL (*p* = 0.003) in the ADVOS group. The relative bilirubin reduction was significantly higher after application of CytoSorb than after treatment with ADVOS (35% (IQR 19,54) vs. 15% (IQR − 11;54), *p* < 0.0001). Regarding patients with ACLF, relative reduction of bilirubin after the first session as well as after the completion of liver support was significantly higher among patients who were treated with CVVHDF and CytoSorb than among those patients who received ADVOS. The relative removal of creatinine and urea nitrogen was significantly higher after ADVOS treatment than after CytoSorb with CVVHDF treatment considering all critically ill patients as well as ACLF patients. Seven-day or in-hospital mortality rates were high among critically ill patients and patients with ACLF in both liver support groups.

**Conclusions:**

Our results showed that CytoSorb and CVVHDF treatment performed better than ADVOS in bilirubin removal among critically ill patients with hyperbilirubinemia caused by acute liver dysfunction and in the subgroup of patients with ACLF. ADVOS was more efficient in eliminating creatinine and urea nitrogen than was CVVHDF with CytoSorb. Additional prospective randomized controlled trials are warranted to investigate the efficacy of hemoperfusion with CytoSorb for liver disease indications among critically ill patients.

**Clinical trial number:**

Not applicable.

**Supplementary Information:**

The online version contains supplementary material available at 10.1186/s12882-025-04342-6.

## Background

Acute impairment of liver function is a common life-threatening condition among critically ill patients admitted to intensive care units (ICUs). The incidence of acute liver dysfunction is as high as 30% among critically ill patients during the course of their illness [1]. The manifestations of acute liver dysfunction among critically ill patients vary but may include acute liver failure (ALF) and acute-on-chronic liver failure (ACLF) [2–4]. Systemic hyperinflammation is involved in the pathogenesis of both ALF and ACLF [5]; however, secondary acquired acute liver failure occurs more often among critically ill patients [6]. Inflammation in the context either of sepsis and multiorgan failure or of exposure to toxic substances predominantly triggers the development of secondary acquired liver failure [3, 7]. Mortality rate among critically ill patients with secondary acquired liver dysfunction reaches up to 11% [8].

Hyperbilirubinemia reflects early hepatic dysfunction among critically ill patients [9]. The serum bilirubin has been established as an appropriate surrogate marker for the assessment of deterioration of excretory liver function. Thus, the serum bilirubin level is a part of several scores [10]. Although bilirubin itself does not exhibit any direct toxic effect on hepatocytes, in several studies the elevation of circulating bilirubin concentrations was shown to correlate with an increase in the risk of mortality among critically ill patients [11, 12]. So far, there is no specific therapy for the management of acute liver dysfunction with hyperbilirubinemia among critically ill patients, and treatment protocols are often center-specific. Liver transplant is the only curative therapy option, but only 4.5% of patients undergo liver transplant because most transplant candidates are considered “too sick to transplant” due to the occurrence of persistent infections and multiorgan failure [13].

Several artificial extracorporeal devices have been tested for replacing hepatic function and bridging patients to liver transplant or recovery [14]. Hemoperfusion using the cytokine adsorber CytoSorb was suggested as a potential method for artificial liver support [3]. This medical device was primarily developed for cytokine removal to control hyperinflammation in septic shock [15]. The adsorber can be implemented in a continuous renal replacement therapy (CRRT) circuit [15]. CytoSorb has a large adsorption capacity because of its surface area of 45,000 qm, and it is reported to eliminate hydrophobic substances with a molecular size of 5 to 55 kD [15]. The molecular weight of bilirubin falls within the aforementioned range of adsorption; thus, the combination of CRRT with CytoSorb absorber can be used for extracorporeal purification of bilirubin from the blood of in critically ill patients with acute kidney injury and hyperbilirubinemia [3, 15–16].

Advanced organ support (ADVOS) is also an artificial liver support tool [17]. It is an extracorporeal multiple organ support system that enables simultaneous replacement of multiple organs, such as liver, kidney, and lungs, with a unified device [17]. ADVOS as part of an advanced hemodialysis system combines three extracorporeal circuits: the extracorporeal blood circuit, the dialysate circuit, and the albumin multicircuit [17]. As a new albumin-dialysis procedure using a recirculating and recyclable albumin-enriched solution, ADVOS allows detoxification of water-soluble and albumin-bound molecules [17].

On the basis of this background information, we decided to perform a retrospective comparison of two artificial systems for liver support: CytoSorb integrated in a continuous veno-venous hemodiafiltration (CVVHDF) circuit, and ADVOS. The aim of this retrospective analysis was to determine and to compare the effects of both liver support modalities in the elimination of bilirubin, liver function and disease severity in a cohort of critically ill patients with acute kidney failure and hyperbilirubinemia caused by diverse forms of acute liver dysfunction, in particular ACLF.

## Methods

### Study population

This monocentric retrospective study compared two approaches for extracorporeal liver support: (1) the combination of the cytokine adsorber CytoSorb (CytoSorbents Europe GmbH, Berlin, Germany) plus CVVHDF, and (2) ADVOS (ADVITOS GmbH, Munich, Germany), in critically ill patients with hyperbilirubinemia in terms of bilirubin elimination and effects on clinical scores and overall survival rates. This study enrolled adult patients with hyperbilirubinemia (total serum bilirubin levels 7 mg/dL or higher) and acute kidney injury requiring CRRT. We included only patients who were exclusively treated with ADVOS or CytoSorb plus CVVHDF. Those patients who received other liver support systems (plasmapheresis, OPAL) before the initiation of ADVOS or CytoSorb plus CVVHDF were excluded. In addition, patients who were treated with several different artificial liver support devices during their ICU stay were also excluded from the analysis. Patients were divided into two groups. One group consisted of 71 critically ill patients who were treated with a combination of CVVHDF and CytoSorb between January 2021 and March 2024 at the University Hospital Essen. The other group consisted of 71 patients who underwent ADVOS therapy during their stay in the ICU of the University Hospital Essen between January 2021 and March 2024. Indications for liver support were ALF or ACLF with hyperbilirubinemia or with hyperbilirubinemia caused by secondary liver dysfunction in the context of sepsis and multiorgan failure. ALF and ACLF were defined in accordance with the European Association for the Study of the Liver guidelines. All included patients received at least one session of treatment with CytoSorb or ADVOS over 24 h. An extracorporeal liver support session was considered to be a single treatment with either CVVHDF plus CytoSorb or ADVOS. Extracorporeal liver support therapy was provided in addition to standard medical care. The type of artificial liver support therapy used, either CytoSorb or ADVOS, and the time point of treatment initiation were decided by the attending treating physician. The use of ADVOS was preferred for patients with ACLF, whereas the combination of CytoSorb with CVVHDF was preferentially used for patients with secondary acquired acute liver dysfunction. The local ethics committee of the University Duisburg-Essen approved this retrospective study (23-11563-BO, 23-11170-BO).

All laboratory variables were determined by standard clinical chemistry tests in the Institute of Laboratory Medicine Essen and were obtained from the laboratory information system within the first 24 h before the initiation of liver support therapy, defined as day 0 (d0), and after 24, 48, and 72 h of treatment, as well as at the end of treatment. Demographic and clinical data were collected from patient information systems by retrospective electronic medical record review. The consent to participate was waived by the local ethics committee of the University Duisburg-Essen due to the retrospective analyses that deal with data obtained from clinical routine.

The clearance of bilirubin and urinary waste products and improvement of the metabolic acidosis were primary outcomes in our study, while paraclinical improvement of the liver function, reduction of the length of the intensive care unit stay, improvement of clinical scoring and overall survival were declared as secondary outcomes.

### Study setting

Patients in the CytoSorb group were treated with the high-flux F60S dialyzer (effective surface area, 1.3-qm; Fresenius, Medical Care AG, Bad Homburg, Germany), and the CytoSorb adsorber was placed upstream of the dialyzer. The duration of a single CytoSorb application was 24 h; the adsorber was exchanged every 24 h. The initial settings for CVVHDF were the following: blood flow of 100 mL/min, dialysate flow of 1000 to 3000 mL/min, and predilution with a 5% glucose solution at 600 to 800 mL/min. The composition of the dialysate for CVVHDF after mixing with 160 mL sodium bicarbonate solution of 8.4% was as follow: sodium 130 mmL/L, potassium 2 mmoL/L, calcium 1.25 mmoL/L, magnesium 0.5 mmoL/L, chloride 122.5 mmoL/L and bicarbonate 13 mmoL/L. Our dialysate contains low amount of bicarbonate due to the anticoagulation with citrate which should be metabolized by the liver to bicarbonate. The entire CVVHDF circuit was exchanged regularly every 72 h. Anticoagulation was performed with regional citrate.

The duration of a single ADVOS session was 24 h. The ADVOS system consisted of three extracorporeal circuits: blood circuit, dialysate circuit, and ADVOS multicircuit [18]. In the extracorporeal blood circuit the patient’s blood was cleaned by two high-flux polyethersulfone dialyzers (SURELYZER PES-190 DH, Nipro D. Med Germany GmbH, Hamburg, Germany) with a 1.9-qm effective surface for each dialyzer [18]. The dialysate circuit and ADVOS multicircuit were necessary for eliminating water-soluble and protein-bound toxins from the patient’s blood [18]. The dialysate contained a mixture of alkaline concentrate (mainly NaOH), an acid concentrate (mainly HCl), osmosis water, and 200 mL of 20% pharmaceutical grade albumin, and it was recirculated with a flow of 800 mL/min in the second circuit [18].

For this recirculating procedure, the dialysate was divided in two paths, in which acidic or basic concentrates were added with a concentrate flow of 160 or 320 mL/min to change the pH and to provoke the release of anionic or cationic albumin-bound substances and, in this way, to regenerate albumin-binding capacity [18]. The pH of the dialysate can be adjusted between 7.2 and 9.0 by modifying the ratio of acidic and basic concentrates [18]. The released anionic or cationic substances were further filtered by convection through two polynephron high-flux dialyzers (ELISIO-13 H, Nipro D. Med Germany GmbH, Hamburg, Germany) with a 1.3-qm effective surface for each dialyzer [18]. Each dialyzer was installed in either an acidic or a basic path of the ADVOS multicircuit [18]. The removed filtrate was continuously replaced by permeate (i.e., osmosis water) and acidic and basic concentrates [18]. ADVOS therapy was conducted at the following initial settings: blood flow, 100 mL/min; concentrate flow, 160 mL/min; dialysate pH, 7.8. Blood anticoagulation was maintained by the application of regional citrate.

### Statistical analysis

Categorical variables were presented as numbers and percentages, and continuous variables were given as medians with interquartile ranges. The two-tailed *χ*^*2*^ test for categorical variables and the Mann-Whitney test for not normally distributed quantitative data were used to detect differences between the CytoSorb and ADVOS groups. The differences between variables obtained at the start of liver support therapy and those obtained at the completion of therapy were analyzed with the Wilcoxon test. Survival was assessed by Kaplan-Meier analysis, and the p values were determined by the log-rank test. All p values are two-tailed, and statistical significance was assumed for p values ≤ 0.05. All statistical analyses were performed with GraphPad Prism version 6 (GraphPad Software, Inc., La Jolla, CA, USA) and IBM SPSS Statistics version 23 (IBM Corp., Armonk, NY, USA).

## Results

### Patient characteristics

Table 1 illustrates patients’ demographic characteristics and laboratory values measured immediately before the initiation of liver support. ACLF was significantly more frequent in the ADVOS group than in the CytoSorb group, whereas secondary acquired acute liver dysfunction occurred significantly more often among patients treated with CytoSorb than among patients treated with ADVOS. Patients from the CytoSorb group stayed significantly longer in an ICU before the initiation of liver support therapy; they also exhibited a significantly higher Sequential Organ Failure Assessment (SOFA) score at baseline than did those patients who underwent ADVOS treatment. Model for End-Stage Liver Disease (MELD) scores at study inclusion were significantly higher among patients in the ADVOS group than among those in the CytoSorb group. With regard to biochemical data, patients in the CytoSorb group exhibited significantly higher baseline levels of liver transaminases, lactate dehydrogenase (LDH), prothrombin time, hemoglobin, C-reactive protein, and procalcitonin. Indeed, baseline partial thromboplastin times (PTT) were higher for patients treated with ADVOS than for those treated with CytoSorb. Baseline total serum bilirubin levels did not differ significantly between the treatment groups.

**Table 1:** Comparison of baseline clinical and laboratory values between 71 patients treated with a combination of cytosorb plus continuous veno-venous hemodiafiltration and 71 patients treated with the ADVOS system. All patients were admitted to the intensive care unit of the university hospital Essen between January 2021 and March 2024 because of critical illness with hyperbilirubinemia and acute kidney injury. Values are presented as medians with interquartile range

Variable	CytoSorb *n* = 71	ADVOS *n* = 71	RR (CI)	*p* value
Age (years)	58 (46–66)	55 (46–64)		0.55
Women, (%)	30 (42)	30 (42)	1.0 (0.7–1.5)	0.99
Number of sessions	3 (2–4)	4 (2–8)		0.07
Duration of CRRT (hours)	72 (48–96)	96 (48–192)		0.07
Dialysis dose (mL/kg/hr)	24 (19–27)	131 (115–151)		**0.0001**
ACLF, (%)	31 (44)	66 (93)	0.5 (0.4–0.6)	**0.0001**
ALF, (%)	5 (7)	5 (7)	1.0 (0.3–3.1)	0.99
Secondary acquired liver dysfunction, (%)	35 (49)	0 (0)	infinity (2.0-infinity)	**0.0001**
Days in ICU before therapy	9 (2–17)	1 (0–7)		**0.0001**
Continous dialysis before therapy, (%)	35 (49)	32 (45)	1.1 (0.8–1.6)	0.61
SOFA (points)	19 (17–20)	18 (16–19)		**0.04**
SAPS II (points)	81 (73–86)	77 (68–84)		0.06
MELD (points)	30 (25–35)	33 (28–37)		**0.04**
**Laboratory values at baseline**				
Bilirubin (mg/dL)	19.9 (13.1–26.9)	13.6 (9.4–23.3)		0.08
ALT (U/L)	88 (41–187)	63 (30–151)		0.06
AST (U/L)	172 (112–408)	139 (79–247)		**0.04**
GGT (U/L)	111 (48–295)	67 (34–157)		**0.009**
LDH (U/L)	511 (318–1610)	347 (250–550)		**0.0006**
Leukocytes (/nL)	15.6 (10.8–22.7)	15.0 (9.7–21.0)		0.42
Hemoglobin (g/dL)	8.9 (7.9–10.1)	7.8 (7.4–9.2)		**0.004**
Platlets (/nL)	87 (42–135)	65 (43–131)		0.26
Prothrombin time (%)	50 (34–68)	33 (24–44)		**0.0001**
PTT (sec)	41 (36–51)	55 (44–73)		**0.0001**
Serum creatinine (mg/dL)	2.1 (1.4–3.3)	2.0 (1.4–3.1)		0.75
Blood urea nitrogen (mg/dL)	57 (35–78)	54 (30–78)		0.81
Lactate (mmol/L)	2.3 (1.5–3.2)	2.6 (1.5–5.3)		0.45
pH	7.39 (7.31–7.48)	7.38 (7.31–7.43)		0.82
Base excess	-0.2 (-3.2-2.1)	-2.2 (-7.2-2.0)		0.20
C-reactive protein (mg/dL)	14 (7–21)	8 (3–13)		**0.0001**
Procalcitonin (ng/mL)	5.2 (2.4–13.3)	2.2 (1.1–5.7)		**0.0001**

ACLF, acute-on-chronic liver failure; ADVOS, advanced organ support; ALF, acute liver failure; ALT, alanine transaminase; AST, aspartate transaminase; CI, confidence interval; CRRT, continuous renal replacement therapy; ICU, intensive care unit; GGT, gamma-glutamyltransferase; L, liter; LDH, lactate dehydrogenase; MELD, Model for End-Stage Liver Disease; PTT, partial thromboplastin time; RR, relative risk; SAPS II, Simplified Acute Physiology Score II; SOFA, Sequential Organ Failure Assessment; U, unit

As indicated in Table 1, there were several differences in baseline characteristic between critically ill patients with hyperbilirubinemia treated with CytoSorb and CVVHDF and those treated with ADVOS that are well explained by different selection of indication for liver support with CytoSorb and ADVOS. ADVOS was exclusively used for patients with ACLF and some few cases of ALF. Indeed, patients having secondary acquired acute liver dysfunction were treated only with combination of CytoSorb and CVVHDF for liver support and ADVOS was never applied in our center for this indication. However, among 71 patients who received CVVHDF with CytoSorb, 31 patients had ACLF. Therefore, we considered a separate analysis of the subgroup of 97 ACLF patients as the best option to create appropriate comparable groups. Table 2 shows clinical and laboratory data at baseline among ACLF patients. Table 2 indicated that baseline characteristics of those ACLF patients who were treated with CytoSorb integrated in CVVHDF were similar to the baseline characteristics of ACLF patients who got ADVOS therapy with the minor exception for aspartate transaminase (AST) and partial thromboplastin time (PTT).

**Table 2:** Comparison of baseline clinical and laboratory values between 31 ACLF patients treated with a combination of cytosorb plus continuous veno-venous hemodiafiltration and 66 ACLF patients treated with the ADVOS system. Values are presented as medians with interquartile range

Variable	CytoSorb *n* = 31	ADVOS *n* = 66	RR (CI)	*p* value
Age (years)	57 (45–64)	55 (46–63)		0.88
Females, (%)	12 (39)	28 (42)	0.91 (0.5–1.5)	0.73
Number of sessions	3 (2–4)	4 (2–8)		0.15
Duration of CRRT (hours)	72 (48–96)	96 (48–198)		0.14
Dialysis dose (mL/kg/hr)	24 (18–28)	130 (117–150)		**0.0001**
Days at ICU before therapy	5 (0–12)	1 (0–7)		0.10
Continous dialysis before therapy, (%)	15 (48)	30 (45)	1.07 (0.7–1.6)	0.79
SOFA (points)	18 (16–20)	18 (16–19)		0.84
SAPS II (points)	81 (67–89)	77 (69–84)		0.40
MELD (points)	30 (27–38)	33 (28–37)		0.56
**Laboratory values at baseline**				
Bilirubin (mg/dL)	17.6 (12.8–31.1)	15.7 (9.2–23.5)		0.10
ALT (U/L)	63 (38–118)	56 (29–138)		0.60
AST (U/L)	139 (94–221)	191 (119–1306)		**0.04**
GGT (U/L)	118 (35–298)	64 (34–162)		0.14
LDH (U/L)	318 (269–440)	340 (247–537)		0.91
Leukocytes (/nL)	15.1 (10.8–23.4)	15.1 (10.0-21.5)		0.86
Hemoglobin (g/dL)	8.1 (7.5–10.0)	7.8 (7.4–9.1)		0.20
Platlets (/nL)	85 (40–149)	63 (41–127)		0.24
Prothrombin time (%)	40 (28–58)	31 (24–43)		0.07
PTT (sec)	44 (33–50)	56 (43–74)		**0.001**
Serum creatinine (mg/dL)	2.2 (1.5–3.5)	2.0 (1.4–3.1)		0.51
Blood urea nitrogen (mg/dL)	52 (30–71)	55 (31–84)		0.60
Lactate (mmol/L)	2.3 (1.5–3.7)	2.8 (1.5–5.4)		0.91
pH	7.38 (7.31–7.43)	7.38 (7.31–7.43)		0.80
Base excess	-2.1 (-5.4-0.9)	-2.6 (-7.2-1.8)		0.89
C-reactive protein (mg/dL)	7.5 (3.5–18.9)	7.8 (2.9–12.7)		0.46
Procalcitonin (ng/mL)	2.7 (1.7–5.9)	2.1 (1.0-5.8)		0.33

ACLF, acute-on-chronic liver failure; ADVOS, advanced organ support; ALT, alanine transaminase; AST, aspartate transaminase; CI, confidence interval; CRRT, continuous renal replacement therapy; ICU, intensive care unit; GGT, gamma-glutamyltransferase; L, liter; LDH, lactate dehydrogenase; MELD, Model for End-Stage Liver Disease; PTT, partial thromboplastin time; RR, relative risk; SAPS II, Simplified Acute Physiology Score II; SOFA, Sequential Organ Failure Assessment; U, unit

### Use of cytosorb was associated with significantly higher clearance of bilirubin than was ADVOS

**Fig. 1:**
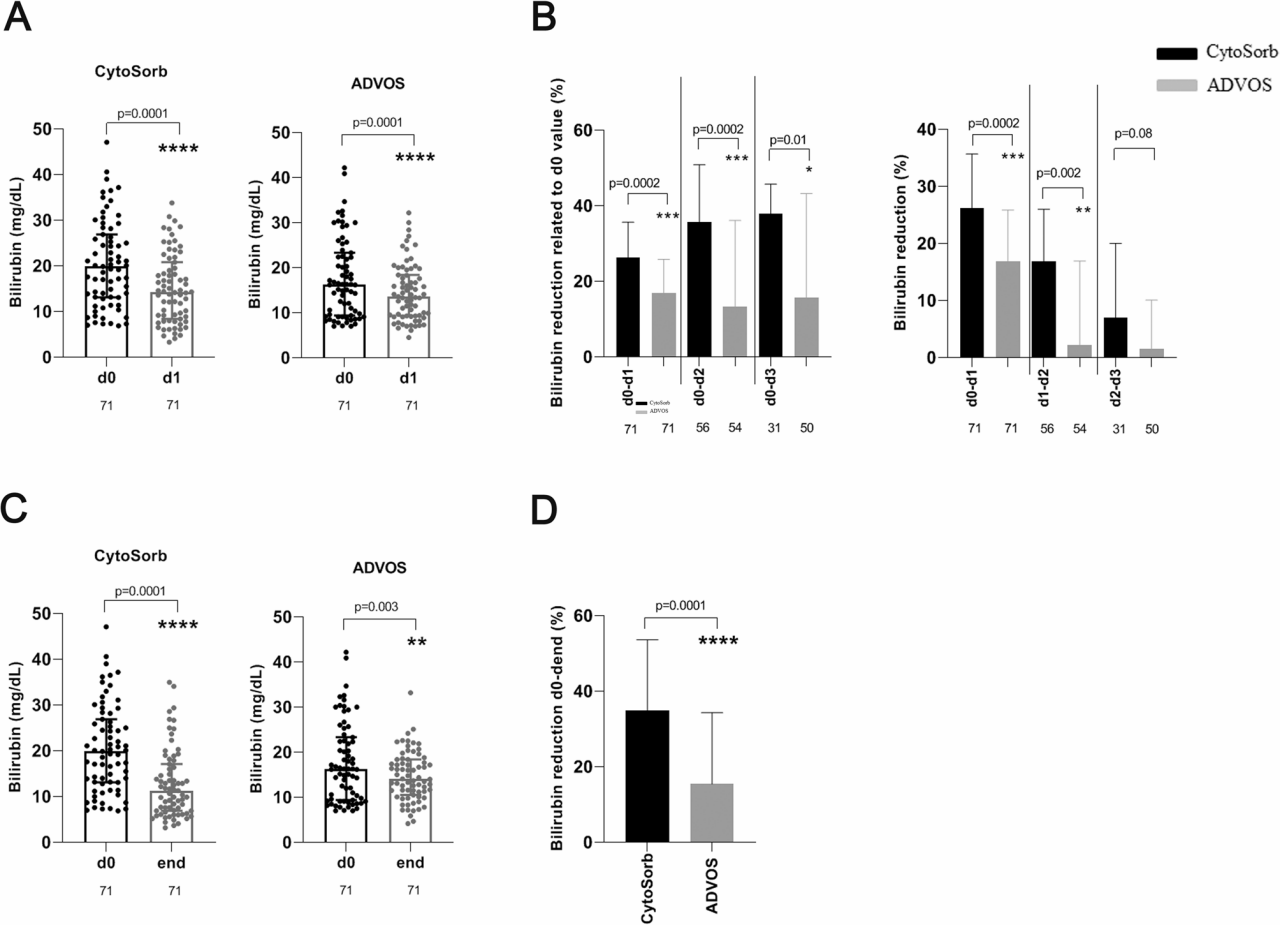
Clearance of total serum bilirubin among 71 critically ill patients treated with a combination of CytoSorb plus continuous veno-venous hemodiafiltration and among 71 critically ill patients treated with the ADVOS system. (**A**) Changes in total bilirubin concentrations after the first single therapy session of 24 h with CytoSorb or with ADVOS. (**B**) Comparison of relative bilirubin reduction (related to baseline [d0] levels or not) during the first three days of treatment with CytoSorb or with ADVOS. (**C**) Changes in total bilirubin concentrations after the completion of therapy with CytoSorb or with ADVOS. (D) Comparison of relative bilirubin reduction achieved at the end of treatment between critically ill patients treated with CytoSorb and those treated with ADVOS. *, *p* < 0.05; **, *p* = 0.01; ***, *p* = 0.001; ****, *p* ≤ 0.0001. ADVOS, advanced organ support; d, day; L, liter

First, we compared the two liver support methods in eliminating total serum bilirubin. Both devices significantly decreased bilirubin concentrations at the end of the first treatment session lasting 24 h (CytoSorb plus CVVHDF, 20 to 14 mg/dL, *p* < 0.0001; ADVOS, 16 to 14 mg/dL, *p* < 0.0001) (Fig. 1A). However, the relative median reduction of total bilirubin levels achieved by CytoSorb within the first 24 h of treatment was significantly higher than that after the first use of ADVOS over 24 h (26% vs. 17%, *p* = 0.0002) (Fig. 1B).

A full course of treatment with either liver support mode significantly reduced total bilirubin levels, but this end-of-treatment reduction was more pronounced in the CytoSorb group (19.9 to 11.3 mg/dl, *p* < 0.0001) than in the ADVOS group (16.3 to 14.0 mg/dL, *p* = 0.003) (Fig. 1C). The median duration of a full course of treatment with CytoSorb was 72 h (range 24–432 h) and with ADVOS 96 h (range 24–1344 h), respectively (*p* = 0.07). As was true for the results of the single session, the elimination of bilirubin after the completion of liver support treatment was significantly higher among patients in the CytoSorb group (35%) than among those in the ADVOS group (15%) (*p* < 0.0001) (Fig. 1D). As shown in Fig. 1B, the highest value of relative bilirubin reduction was achieved after the first day of treatment with either liver support option. After 48 and 72 h of treatment, both liver support modalities exhibited a decrease in the elimination capacity of bilirubin among critically ill patients who survived longer than one day (Fig. 1B). However, the relative removal of bilirubin by CytoSorb was significantly higher than the relative removal by ADVOS at all time points of treatment (Fig. 1B).

**Fig. 2:**
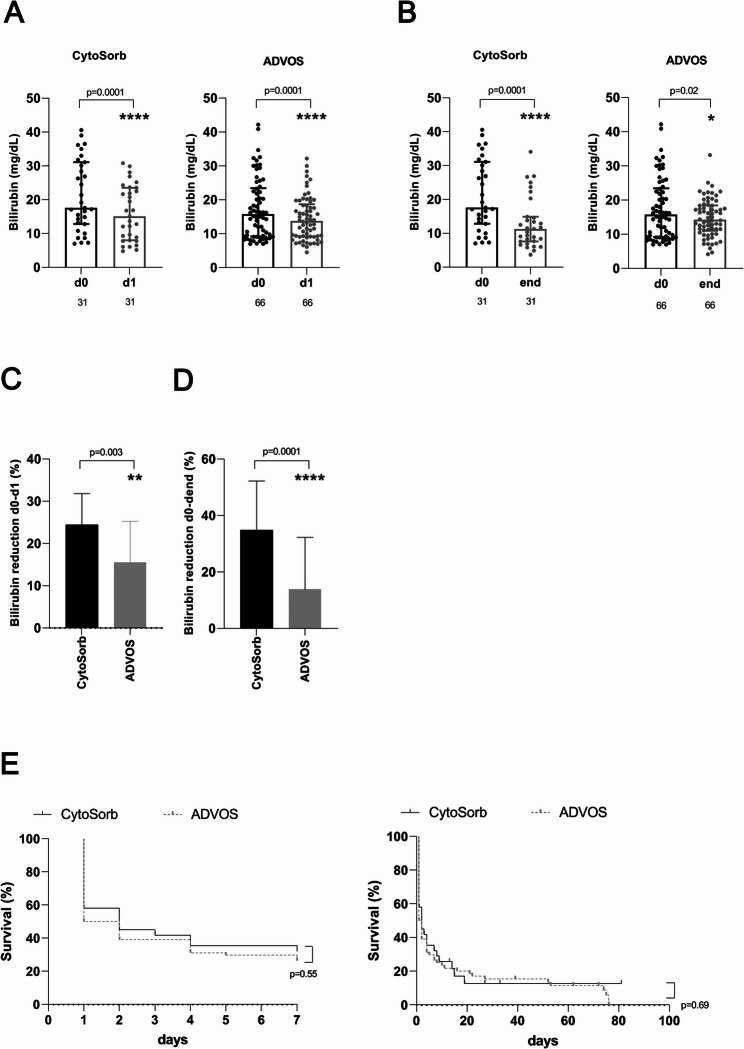
Effects on biochemical and clinical outcomes of adjuvant extracorporeal liver support treatment for critically ill patients with acute-on-chronic liver failure (ACLF). Patients were treated either with a combination of CytoSorb plus continuous veno-venous hemodiafiltration or with the ADVOS system. Shown are changes in total bilirubin concentrations after the first single therapy session (**A**) and after the completion of therapy (**B**) with CytoSorb or with ADVOS. Also shown are comparisons of relative bilirubin reduction achieved after the first single therapy session (**C**) and at the end of treatment (**D**) between critically ill patients treated with CytoSorb and those treated with ADVOS. (**E**) Short-term seven-day mortality rates and in-hospital mortality rates for critically ill patients with ACLF treated with CytoSorb or with ADVOS. *, *p* < 0.05; **, *p* = 0.01; ****, *p* ≤ 0.0001. ADVOS, advanced organ support; d, day; L, liter

Next, we separately analyzed the subgroup of 97 patients for whom ACLF was the cause of liver dysfunction with hyperbilirubinemia (Fig. 2). The initial total bilirubin levels (CytoSorb, 17.6 mg/dL; ADVOS, 15.7 mg/dL; *p* = 0.1) and the number of sessions (CytoSorb, 3 sessions; ADVOS, 4 sessions; *p* = 0.2) were similar for patients in both groups (Table 2). The median duration of the liver support (72 (range 24–240 h) vs. 96 h (range 24–1344 h); *p* = 0.15), as well as the median duration of continuous renal replacement therapy (72 (range 24–576 h) vs. 96 h (range 24–1344 h); *p* = 0.14) were also comparable in both groups (Table 2). The results achieved by the analysis of bilirubin clearance for this subgroup were similar to those achieved by analysis of the entire cohort (Fig. 2A-D). Again, the relative reductions in total bilirubin levels after the first single session (Fig. 2C) and at the end of treatment (Fig. 2D) were significantly higher in the CytoSorb group than in the ADVOS group.

### Changes in biochemical variables during liver support with cytosorb versus ADVOS

Changes between pretreatment and posttreatment laboratory values in the two patient groups are depicted in Table 3. The full course of treatment with CytoSorb was associated with a significant decrease in gamma-glutamyl transferase (GGT) activity, in prothrombin time, and in creatinine, blood urea nitrogen, platelet count, hemoglobin, C-reactive protein, and procalcitonin levels, and with a significant increase of in PTT and lactate levels (Table 3). Treatment with ADVOS led to a significant decrease in levels of creatinine, blood urea nitrogen, prothrombin time, platelet count, hemoglobin, and C-reactive protein and to a significant elevation of transaminase activity, lactate dehydrogenase (LDH) activity, PTT, lactate levels, and pH (Table 3). Transfusion of platelets was necessary in 28 (39%) patients in the CytoSorb group whereas platelet transfusion were conducted in 25 (35%) patients in the ADVOS group (relative risk (CI) 1.12 (0.7–1.7); *p* = 0.60). The intergroup comparison showed a significantly higher relative reduction of serum creatinine and urea nitrogen among patients treated with ADVOS than among those treated with the combination of CVVHDF and CytoSorb (Table 4). A percentage increase in posttreatment transaminase activity and LDH activity occurred in the ADVOS group but not in the CytoSorb group (Table 4). The relative reduction of procalcitonin levels was significantly higher in the CytoSorb group than in the ADVOS group, a finding suggesting that ADVOS treatment did not influence procalcitonin levels (Table 4).

**Table 3:** Changes in absolute values in relevant biochemical variables after adjuvant extracorporeal liver support treatment of critically ill patients with hyperbilirubinemia and acute kidney injury. Patients were treated either with a combination of cytosorb plus continuous veno-venous hemodiafiltration or with the ADVOS system. Values are presented as medians with interquartile range

	CytoSorb			ADVOS		
Variable	Pretreatment (d0)	Posttreatment (end of therapy)	*p* value	Pretreatment (d0)	Posttreatment (end of therapy)	*p* value
ALT (U/L)	88 (41–187)	89 (44–330)	0.45	63 (30–151)	84 (43–484)	**0.0002**
AST (U/L)	172 (112–408)	172 (101–855)	0.17	139 (79–247)	208 (119–2122)	**0.0001**
GGT (U/L)	111 (48–295)	96 (38–253)	**0.0002**	67 (34–157)	72 (32–134)	0.06
LDH (U/L)	511 (318–1610)	511 (346–1569)	0.95	347 (250–550)	587 (318–2207)	**0.0001**
Leukocytes (/nL)	15.6 (10.8–22.7)	16.2 (11.6–22.8)	0.87	15.0 (9.7–21.0)	18.3 (14.5–23.2)	0.10
Hemoglobin (g/dL)	8.9 (7.9–10.1)	8.4 (7.3–9.8)	**0.0001**	7.8 (7.4–9.2)	7.7 (7.2–8.7)	**0.02**
Platlets (/nL)	87 (42–135)	58 (35–101)	**0.0001**	65 (43–131)	37 (15–81)	**0.0001**
Prothrombin time (%)	50 (34–68)	44 (21–61)	**0.0002**	33 (24–44)	27 (15–40)	**0.0001**
PTT (sec)	41 (36–51)	56 (45–94)	**0.0001**	55 (44–73)	75 (49–170)	**0.0001**
Serum creatinine (mg/dL)	2.1 (1.4–3.3)	1.6 (1.0-2.3)	**0.0001**	2.0 (1.4–3.1)	1.2 (0.8–1.6)	**0.0001**
Blood urea nitrogen (mg/dL)	57 (35–78)	40 (25–58)	**0.0001**	54 (30–78)	23 (16–33)	**0.0001**
Lactate (mmol/L)	2.3 (1.5–3.2)	2.6 (1.6–5.7)	**0.0003**	2.6 (1.5–5.3)	3.6 (1.8–11.0)	**0.001**
pH	7.39 (7.31–7.48)	7.38 (7.31–7.44)	0.43	7.38 (7.31–7.43)	7.43 (7.32–7.47)	**0.03**
Base excess	-0.2 (-3.2-2.1)	-0.9 (-5.8-2.3)	0.17	-2.2 (-7.2-2.0)	1.1 (-7.5-5.5)	0.06
C-reactive protein (mg/dL)	14 (7–21)	12 (5–17)	**0.003**	8 (3–13)	6 (3–10)	**0.03**
Procalcitonin (ng/mL)	5.2 (2.4–13.3)	2.7 (1.3–6.5)	**0.0001**	2.2 (1.1–5.7)	2.0 (1.0-4.8)	0.16

ADVOS, advanced organ support; ALT, alanine transaminase; AST, aspartate transaminase; CRP, C-reactive protein; d, day; GGT, gamma-glutamyltransferase; L, liter; LDH, lactate dehydrogenase; PTT, partial thromboplastin time

**Table 4:** Relative reductions in relevant biochemical variables after adjuvant extracorporeal liver support treatment of critically ill patients with hyperbilirubinemia and acute kidney injury. Patients were treated either with a combination of cytosorb plus continuous veno-venous hemodiafiltration or with the ADVOS system

Median relative reduction (%)	CytoSorb *n* = 71	ADVOS *n* = 71	*p* value
ALT	-3.8	-33.3	**0.01**
AST	3.3	-66.7	**0.002**
GGT	20.5	7.7	0.24
LDH	5.5	-48.5	**0.001**
Leukocytes	-1.2	-11.4	0.33
Hemoglobin	7.9	4.0	0.54
Platlets	32.5	38.3	0.23
Prothrombin time	18.6	20.0	0.57
PTT	-28.5	-47.6	0.73
Serum creatinine	22.0	44.8	**0.0007**
Blood urea nitrogen	22.0	52.0	**0.0001**
Lactate	-28.6	-50.0	0.55
pH	0.07	-0.5	**0.02**
Base excess	32.9	75.6	0.80
C-reactive protein	21.4	17.5	0.69
Procalcitonin	50.2	16.6	**0.001**

ADVOS, advanced organ support; ALT, alanine transaminase; AST, aspartate transaminase; CRP, C-reactive protein; d, day; GGT, gamma-glutamyltransferase; L, liter; LDH, lactate dehydrogenase; PTT, partial thromboplastin time

As indicated in Supplementary Tables 1 through 3, an additional focus on the subgroup of ACLF patients showed alterations in relevant laboratory values similar to those achieved with the entire cohort. In summary, ADVOS treatment led to a larger relative reduction of serum creatinine und blood urea nitrogen than did CytoSorb treatment, but it was related to an additional deterioration of liver function with a corresponding additional increase in liver enzyme activity during the treatment course (Supplementary Table 3). In contrast to ADVOS therapy, CytoSorb treatment led to stable liver function values and a significant decrease in procalcitonin concentrations at the end of treatment (Supplementary Table 3). Among patients with ACLF who were treated with combination of CVVHDF and CytoSorb 6 (19%) patients required platelet transfusion while among ACLF patients who underwent ADVOS therapy platelet transfusion was needed in 25 (38%) patients (group (relative risk (CI) 0.51 (0.2–1.1); *p* = 0.07).

### Clinical scoring and short-term mortality under cytosorb and ADVOS

**Fig. 3:**
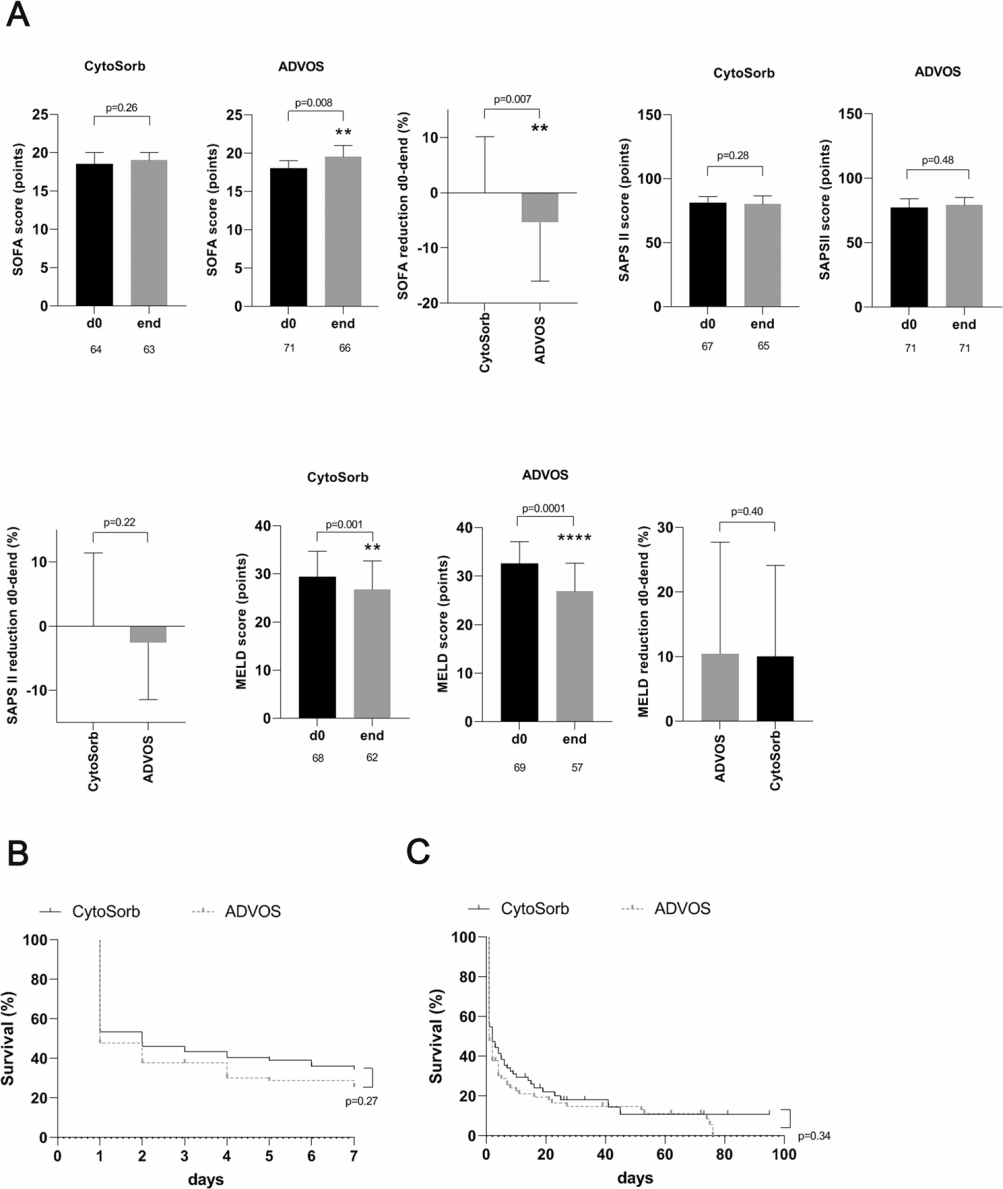
Effects on clinical outcomes of adjuvant extracorporeal liver support treatment of critically ill patients with hyperbilirubinemia and acute kidney injury. Patients were treated either with a combination of CytoSorb plus continuous veno-venous hemodiafiltration or with the ADVOS system. (**A**) Alterations in absolute values and relative reductions in prognostic clinical scores (SOFA, SAPS II, and MELD scores) at the end of treatment with CytoSorb or with ADVOS. Short-term seven-day mortality rates (**B**) and in-hospital mortality rates (**C**) for critically ill patients with hyperbilirubinemia treated either with CytoSorb or with ADVOS. *, *p* < 0.05; **, *p* = 0.01; ***, *p* = 0.001; ****, *p* ≤ 0.0001. ADVOS, advanced organ support; d, day; MELD, Model for End-Stage Liver Disease; SAPS II, Simplified Acute Physiology Score II; SOFA, Sequential Organ Failure Assessment

With respect to prognostic clinical scores, in the CytoSorb group we found no changes between pretreatment and posttreatment SOFA scores or Simplified Acute Physiology Score II (SAPS II) scores (Fig. 3A). In the ADVOS group SOFA scores further increased and SAPS II scores remained unchanged after the completion of adjuvant liver support (Fig. 3A). We found a significant improvement in MELD scores after treatment with both liver support devices (Fig. 3A). Comparison of relative reduction of clinical scores between the two liver support systems showed no differences between the groups in SAPS II and MELD scores (Fig. 3A). Compared to patients treated with CytoSorb, patients in the ADVOS group experienced a significant worsening of SOFA scores during the posttreatment period (Fig. 3A).

For the subgroup of ACLF patients, none of the three clinical scores was influenced by CytoSorb therapy (Supplementary Table 1). ADVOS led to a further elevation in the SOFA score and a decrease in the MELD score after treatment (Supplementary Table 2). Results of the relative changes in clinical scores among critically ill ACLF patients completely reproduced the results achieved for the entire cohort (Supplementary Table 3).

We examined short-term mortality among critically ill patients with hyperbilirubinemia due to acute liver dysfunction who received the two different liver support approaches (Fig. 3B-C). There were no significant differences between the CytoSorb and the ADVOS group in seven-day or in-hospital mortality rates (Fig. 3B). In-hospital mortality rates reached 88% for both devices (Fig. 3C). Separate analysis of ACLF patients for each liver support method showed that seven-day and in-hospital mortality rates were similar to those obtained for the entire study population; there were no significant differences between the two tested liver support devices (Supplementary Fig. 1E).

## Discussion

Our retrospective study compared the effects of two artificial liver support systems, CytoSorb and ADVOS, applied in addition to standard treatment for critically ill patients with hyperbilirubinemia of ≥ 7 mg/dL. We found a more pronounced reduction of total serum bilirubin by CytoSorb integrated into CVVHDF circuit than by ADVOS therapy as early as after one therapy session and also after the completion of treatment. ADVOS was significantly more effective in eliminating water-soluble molecules such as serum creatinine and blood urea nitrogen and in correcting pH alterations than was CytoSorb integrated into a CVVHDF circuit. Platelet counts and hemoglobin and C-reactive protein levels decreased significantly after therapy with both devices, whereas only CytoSorb resulted in a significant decrease in procalcitonin levels. Neither device improved other laboratory liver-function parameters, such as transaminases or plasmatic coagulation. SOFA and SAPS II scores also did not improve after treatment with either liver support method. Our analysis showed high post treatment mortality among critically ill patients treated with the two different liver support modalities. The results of laboratory and clinical outcomes tests obtained from a separate analysis of the subgroup of ACLF patients were similar to those for the entire cohort.

The evidence of the effectiveness of CytoSorb in removal of total serum bilirubin of patients with hyperbilirubinemia is still scarce and is based on case presentations, three observational studies, and one registry analysis [3]. We detected a median decrease of total bilirubin of 6 mg/dL as early as after the first individual session of CytoSorb. The median relative reduction of bilirubin levels after the full course of treatment with CytoSorb was 35%. These findings are consistent with existing reports [3, 16]. Most of the previous studies and case series used retrospectively collected data and involved small sample sizes [3, 16, 19–20]. The CytoSorb International Registry presented by Ocskay et al. analyzed a total of 109 patients who were treated for the liver indication with hyperbilirubinemia; this is the largest dataset obtained in a prospective multicenter fashion [16]. Both Ocskay’s study and the pooled data metaanalysis conducted by Turan et al. reported a mean difference of 5 mg/dL between pretreatment and posttreatment bilirubin levels [3, 16]. The studies of Scharf et al. and Geimel et al. found that the use of CytoSorb led to a median relative reduction in bilirubin levels ranging between 23% and 32%, a finding very similar to our results [19, 20]. In a subset of patients with ACLF, Haselwanter et al. showed a much higher reduction in the relative bilirubin level of 48% after CytoSorb treatment [21]. However, our current study found a median relative reduction rate in the total bilirubin level of 35% among the entire study population as well as among the subgroup of patients with ACLF at the end of CytoSorb therapy. It is worth noting that Geimel et al. observed a relative bilirubin reduction of 32% within the first 6 h of therapy with CytoSorb, whereas a relative bilirubin decrease of only 4% occurred after 6 h [20]. This rapid decline of relative bilirubin clearance during treatment with CytoSorb suggests saturation of the CytoSorb adsorber [20]. Alternatively, Geimel and colleagues hypothesized that the release of bilirubin from the adsorber into the blood circulation during the procedure might explain this phenomenon [20]. In the current study settings the CytoSorb adsorber was replaced after 24 h. Therefore, we assume that because of the potential saturation of the CytoSorb adsorber we may have underestimated the bilirubin elimination, and this underestimation may have prevented us from achieving the same high relative reduction rates as those demonstrated by Haselwanter et al. [21]. In contrast to our study, which already included patients with hyperbilirubinemia of ≥ 7 mg/dl, all other studies enrolled patients with slightly higher total bilirubin concentrations of more than 10 mg/dL [3, 16, 19–21]. Thus, higher concentration gradients may have enabled a faster and more efficient bilirubin removal attributed to the concentration-dependent bilirubin elimination manner of CytoSorb.

We saw a markedly higher relative reduction of total bilirubin levels among 71 critically ill patients and 31 ACLF patients treated with Cytosorb than among 71 critically ill patients and 66 ACLF patients treated with ADVOS. Reports on bilirubin removal achieved by ADVOS are rare to date, mostly deriving from real-life treatment experience [17, 18–22]. Fuhrmann et al. reported only a moderate median decrease in bilirubin levels from 6.9 to 6.5 mg/dL among 18 patients treated with ADVOS in the context of a multicenter registry [17]. A case series of 34 critically ill patients treated with ADVOS found a relative bilirubin reduction of 17%, a finding comparable to our finding of a relative reduction of 15% at the end of ADVOS therapy [17]. Additionally, a concentration-dependent removal of bilirubin was previously described [23]. Scharf et al. compared CytoSorb and ADVOS administered to patients admitted to an ICU with acute liver dysfunction; this group identified an equivalent relative bilirubin reduction of 23% for both liver support systems [19]. However, for this comparison only 6 patients were treated with ADVOS, whereas 33 patients were treated with CytoSorb [19]. The study by Scharf and colleges involved a homogenous patient cohort with secondary acquired acute liver dysfunction [19]. Indeed, in the present study we recruited critically ill patients with hyperbilirubinemia caused by various types of liver function impairment without differentiation between ALF, ACLF, or secondary acquired acute liver dysfunction associated with critical illness and multiorgan failure. Thus, our study population was heterogeneous, with different diagnostic and prognostic characteristics in the two treatment groups. To counteract the aforementioned problem, we focused on the subgroup of patients with ACLF, who exhibited comparable baseline characteristics and not on patients with secondary acquired acute liver dysfunction, who were lacking in our ADVOS treatment group. The analysis of the subgroup of ACLF patients for the CytoSorb and ADVOS device provided results that were in line with observations achieved in the entire cohort. However, most of the patients in the ADVOS group were admitted to the ICU with advanced ACLF stages for which treatment with ADVOS and subsequent bilirubin clearance may be less effective because of the progression of irreversible multiorgan failure and persistent release of bilirubin that may provoke a permanent supply of bilirubin or an additional increase instead of a decrease in bilirubin levels. Studies of the use of CytoSorb and other available liver support modes in separate groups sharing the same indication for liver support and the same entity of liver dysfunction may be helpful in defining which specific types of acute liver dysfunction may profit from CytoSorb.

Dhokia et al. and Scharf et al. found a significant improvement in liver function tests including a decrease in liver transaminase and GGT activity after the completion of CytoSorb therapy [19, 24]. Additionally, Popescu et al. noted that LDH and ammonia levels decreased after CytoSorb treatment with no significant changes in transaminase activity [25]. Because the molecular size of these aforementioned liver variables exceeds CytoSorb’s maximum pore size of 17 kD, it is unlikely that hemoadsorption by CytoSorb is directly responsible for the decrease in the activity of liver transaminases, GGT, and LDH [19]. It is conceivable that the improvement in these clinical values is related to the potential improvement and recovery of liver function under liver support therapy [19]. To resolve this issue, the levels of these substances of interest should be measured upstream and downstream of the CytoSorb adsorber so that clearance by hemoadsorption can be assessed. ADVOS therapy cannot directly remove and modulate liver enzymes [17, 18]. Our data on the course of liver transaminases obtained from the entire study cohort should be interpreted with caution and the observed differences in relative change of transaminases between the CytoSorb and ADVOS group might be produced by baseline differences in several laboratory values between the two groups. However, regarding ACLF patients with comparable levels of ALT, GGT and LDH at baseline between the two treatment groups, we saw no improvement of ALT and LDH levels at the end of treatment with both devices that may refer to irreversible progression of ACLF disease with further deterioration of the liver function despite of the application of liver support.

The significant drop of in platelet counts were of similar extent for both tested liver support methods in our study. A number of other studies have documented depletion of platelets as an adverse effect of CytoSorb use; the degree of this depletion may depend on multiple factors, such as the activation of platelets by the extracorporeal CRRT circuit and the aggregation of platelets within the CytoSorb adsorber or the CRRT dialyzer [20, 21–26]. A reduction in the platelet count was reported to occur during ADVOS therapy [17]. But the observed decrease of platelet counts in our study may also reflect further damage of liver tissue and loss of liver function, especially among patients with advanced ACLF.

Patients in the CytoSorb group experienced a stronger reduction of hemoglobin concentrations after therapy than did those in the ADVOS group. Popescu et al. found similar hemoglobin changes after CytoSorb treatment [25]. Erythrocyte entrapment, destruction within the CytoSorb adsorber or the CRRT dialyzer, and an accompanying phenomenon for critical illness or frequent blood withdrawal during CVVHDF in the ICU should be discussed as potential causes [25, 27–29].

The current study showed that ADVOS therapy was superior to the combination of CVVHDF plus CytoSorb in clearing creatinine and blood urea nitrogen levels, and in improving acidosis. Few existing case series have shown that critically ill patients treated with ADVOS developed successful reduction of creatinine and blood urea nitrogen levels [17, 18–22]. The integration of two high-flux dialyzers with a large surface area of 1.9 qm for each dialyzer into the blood circuit of the ADVOS machine provides an advantage over conventional CVVHDF which uses only one dialyzer. This difference may have substantially contributed to the greater elimination of water-soluble toxins in the ADVOS group than in the CytoSorb group [17, 18]. However, the same blood flow of 100 mL/min was applied in both liver support modalities. Otherwise, we observed a slight trend toward higher median duration of CRRT using ADVOS lasting 96 h in comparison to the median duration of continuous hemodialysis treatment among patients in the CytoSorb group lasting only 72 h, that might partly explain a better removal of serum creatinine and blood urea nitrogen under ADVOS than under CytoSorb with CVVHDF. Thanks to the adjustable dialysate composition in the ADVOS system, a relevant pH increase can be achieved, and severe metabolic disorders refractory to conventional CCRT can be corrected [17, 18]. Previous real-world clinical experience reports indicated that the pH value of the dialysate can be set between 7.2 and 9.0; this setting allows an automatic modification of the dialysate composition according to the amount of acid and basic concentrate being supplied. It also allows adaptation of metabolic control on the individual patient’s needs [17, 18].

Although CytoSorb was significantly better than ADVOS in reducing bilirubin levels, reduction capability of CytoSorb treatment was not associated with ameliorated seven-day or in-hospital survival rates or with improvement in clinical scores such as SOFA and SAPS II. SOFA and SAPS II scores did not decrease in either liver support group after the full course of treatment, probably because these critically ill patients with hyperbilirubinemia were at a late stage of advanced liver disease and multiorgan failure at the time of treatment. The observations of prognostic clinical scores are consistent with the findings of Popescu et al. [25], who maintained that SOFA scores did not change significantly after treatment with CytoSorb or MARS [25]. Like us, they observed a decrease in MELD scores in the CytoSorb group [25]. A reduction in posttreatment MELD scores was documented for both liver support devices in our study; this improvement of MELD scores was artificial and it was attributed to the removal of bilirubin and creatinine by both extracorporeal therapies; these values are included in the quantification of the MELD score.

In-hospital mortality rates were comparably high in both groups, at 88%. In agreement with our findings, Scharf et al. described in-hospital mortality rate of 82% for their cohort of critically ill patients with secondary acquired acute liver dysfunction [19]. Indeed, the CytoSorb International Registry analysis by Ocskay et al. found a lower in-hospital mortality rate of only 60% among 109 critically ill patients treated with CytoSorb for a liver indication [16]. In our opinion, the very high mortality rates observed in our study were probably the result of the inclusion of extremely ill patients with advanced disease state in our study population, as reflected by high MELD, SOFA, and SAPS II scores in both analyses [19]. The present study did not provide an appropriate control group composed of critically ill patients with hyperbilirubinemia who were not treated with a liver support device in addition to standard medical treatment; the absence of a control group complicates the estimation of the real effects of CytoSorb and ADVOS on mortality rates. However, several aspects may play a role in explaining why long-term effects of CytoSorb on mortality rates are not achieved despite a positive effect on total serum bilirubin levels. Hemoadsorption using CytoSorb is not able to completely restore all liver function; it is only an adjuvant treatment option that supports liver function, but does not eliminate the cause of liver failure, or cure acute liver dysfunction. The acute liver dysfunction indicated by hyperbilirubinemia is only one component of a complex disease state and is often accompanied by multiorgan failure. Numerous other factors are involved in the death of critically ill patients with multiorgan failure in ICUs, and one liver support device cannot manage all of these factors. On the other hand, achieving a relevant prognostic improvement in our study population was difficult due to the fact that critically ill patients in both treatment groups mostly exhibited advanced liver dysfunction and multiorgan failure; it was impossible to reverse these conditions by using adjuvant liver support therapies that might be provided too late. Thus, our study results emphasize the urgent need for the application of CytoSorb, ADVOS, or another liver support system at early disease stages to prevent disease progression.

We are aware of several limitation of our study. The data were obtained retrospectively in clinical routines from a single center. Hence, missing laboratory values or slight deviation in time points cannot be excluded. Because the study did not include an appropriate control group, it is problematic to account for spontaneous changes in characteristic variables of liver function and to judge the advantages in survival and recovery from acute liver dysfunction. Our study may also involve selection bias because of heterogeneity in diagnosis and prognosis in both treatment groups and deviant composition in terms of forms of acute liver dysfunction in the CytoSorb group as compared with the ADVOS group; this bias may have been induced because the choice of a suitable liver support device was based on the decision and opinion of an attending physician in our institution, because consensus and protocols on this issue are lacking so far. In the case of ACLF, the use of ADVOS instead of CytoSorb was favored and recommended in our center. In the CytoSorb group secondary acquired acute liver dysfunction was the most common underlying entity of liver failure, whereas in the ADVOS group secondary acquired acute liver dysfunction was missing. Significant differences in clinical and biochemical baseline features between the CytoSorb group and the ADVOS group may also have induced selection bias. Critically ill patients with hyperbilirubinemia who were treated with combination of CVVHDF with CytoSorb stayed significantly longer at intensive care unit before the initiation of liver support than those patients who were treated afterwards with ADVOS that might have caused additional bias. Nevertheless, compared with a number of other recent studies and case series, the current study involved a large patient number and dataset in both liver support therapy groups.

## Conclusions

In conclusion, both liver support devices, CytoSorb and ADVOS, efficiently reduced bilirubin levels among critically ill patients with hyperbilirubinemia caused by various forms of acute liver dysfunction and among critically ill patients with ACLF. Although CytoSorb use led to a significantly more extensive relative reduction of total serum bilirubin levels than did ADVOS, ADVOS therapy was associated with better elimination of water-soluble substances such as creatinine and blood urea nitrogen and with a better rebalancing of acid abnormalities than was CytoSorb. Neither of the liver support modalities improved liver function and short-term mortality rates of critically ill patients with hyperbilirubinemia or ACLF patients were comparable between the two extracorporeal liver support approaches. Artificial liver support devices such as ADVOS are time- and cost-intensive, and their use is complex, requiring experienced healthcare personnel [17, 18]. In contrast, CytoSorb as a liver support approach is cheaper, easier to handle, and readily available for installation in conventional hemodialysis circuits [16]. In addition, our findings support the opinion that CytoSorb has beneficial effects on bilirubin elimination and is not disadvantageous compared to ADVOS. Therefore, the CytoSorb adsorber may be a promising tool for treating critically ill patients with acute liver dysfunction and hyperbilirubinemia in small centers. Otherwise, the combination of ADVOS and CytoSorb incorporates the advantages of both devices, such as bilirubin clearance, elimination of water-soluble toxins, and correction of metabolic disorders, this combination merits investigation in ongoing studies involving critically ill patients with acute liver dysfunction.

## Electronic supplementary material

Below is the link to the electronic supplementary material.

Supplementary Material 1

## Data Availability

All data generated or analyzed during this study are included in this published article and its supplementary information files.

## Abbreviations

ACLF: Acute-on-chronic liver failure
ADVOS: Advanced organ support
ALF: Acute liver failure
ALT: Alanine transaminase
AST: Aspartate transaminase
CI: Confidence interval
CRP: C-reactive protein
CRRT: Continuous renal replacement therapy
CVVHDF: Continuous veno-venous hemodiafiltration
d: day
ICU: Intensive care unit
GGT: Gamma-glutamyltransferase
L: Liter
LDH: Lactate dehydrogenase
MARS: Molecular Adsorbent Recirculating System
MELD: Model for End-Stage Liver Disease
PTT: Partial thromboplastin time
RR: Relative risk
SAPS II: Simplified Acute Physiology Score II
SOFA: Sequential Organ Failure Assessment
U: Unit
urea-N: Urea nitrogen
