# Supplementary material for: Artificial liver support with Cytosorb and continuous veno-venous hemodiafiltration versus advanced organ support (ADVOS) for critically ill patients with hyperbilirubinemia and acute-on-chronic liver failure (ACLF)
Source: BMC Nephrol. 2025 Aug 4;26:432. doi: 10.1186/s12882-025-04342-6 (PMC12323257; doi:10.1186/s12882-025-04342-6)
Supplement: Supplementary file 1 — Supplementary Material 1 [file 12882_2025_4342_MOESM1_ESM.docx]

**Supplementary Tables**

**Supplementary Table 1.** Alterations in relevant biochemical variables and prognostic clinical scores after adjuvant extracorporeal liver support treatment of critically ill patients with hyperbilirubinemia and acute kidney injury caused by acute-on-chronic liver failure (ACLF). Patients were treated with a combination of CytoSorb plus continuous veno-venous hemodiafiltration. Absolute pretreatment and posttreatment values were compared, and all values are presented as medians.

| **Variable** | **Pretreatment (d0)** | **Posttreatment (end of therapy)** | **p value** |
| --- | --- | --- | --- |
| ALT (U/L) | 63 | 60 | 0.80 |
| AST (U/L) | 139 | 150 | 0.09 |
| GGT (U/L) | 118 | 103 | **0.009** |
| LDH (U/L) | 318 | 353 | 0.55 |
| Leukocytes (/nL) | 15.1 | 19.7 | **0.03** |
| Hemoglobin (g/dL) | 8.1 | 8.1 | **0.0004** |
| Platlets (/nL) | 85 | 58 | **0.004** |
| Prothrombin time (%) | 40 | 32 | **0.02** |
| PTT (sec) | 44 | 63 | **0.0001** |
| Serum creatinine (mg/dL) | 2.2 | 1.7 | **0.0001** |
| Blood urea nitrogen (mg/dL) | 52 | 33 | **0.0005** |
| Lactate (mmol/L) | 2.3 | 2.8 | **0.02** |
| pH | 7.38 | 7.37 | 0.42 |
| Base excess | -2.1 | -2.5 | 0.42 |
| C-reactive protein (mg/dL) | 7.5 | 8.5 | 0.83 |
| Procalcitonin (ng/mL) | 2.7 | 1.6 | **0.003** |
|  |  |  |  |
| SOFA (points) | 18 | 19 | 0.31 |
| SAPS II (points) | 81 | 78 | 0.53 |
| MELD (points) | 0 | 28 | 0.11 |

ALT, alanine transaminase; AST, aspartate transaminase; GGT, gamma-glutamyltransferase; L, liter; LDH, lactate dehydrogenase; MELD, Model for End-Stage Liver Disease; PTT, partial thromboplastin time; SAPS II, Simplified Acute Physiology Score II; SOFA, Sequential Organ Failure Assessment.

**Supplementary Table 2.** Alterations in relevant biochemical variables and prognostic clinical scores after adjuvant extracorporeal liver support treatment of critically ill patients with hyperbilirubinemia and acute kidney injury caused by acute-on-chronic liver failure (ACLF). Patients were treated with ADVOS. Absolute pretreatment and posttreatment values were compared, and all values are presented as medians.

| **Variable** | **Pretreatment (d0)** | **Posttreatment (end of therapy)** | **p value** |
| --- | --- | --- | --- |
| ALT (U/L) | 56 | 80 | **0.0002** |
| AST (U/L) | 137 | 191 | **0.0001** |
| GGT (U/L) | 64 | 64 | 0.08 |
| LDH (U/L) | 340 | 578 | **0.0001** |
| Leukocytes (/nL) | 15.1 | 18.4 | 0.14 |
| Hemoglobin (g/dL) | 7.8 | 7.6 | **0.02** |
| Platlets (/nL) | 63 | 37 | **0.0001** |
| Prothrombin time (%) | 31 | 28 | **0.0001** |
| PTT (sec) | 56 | 63 | **0.02** |
| Serum creatinine (mg/dL) | 2.0 | 1.2 | **0.0001** |
| Blood urea nitrogen (mg/dL) | 55 | 24 | **0.0001** |
| Lactate (mmol/L) | 2.8 | 3.3 | **0.002** |
| pH | 7.38 | 7.43 | **0.03** |
| Base excess | -2.6 | 1.7 | **0.03** |
| C-reactive protein (mg/dL) | 8 | 9 | 0.15 |
| Procalcitonin (ng/mL) | 2.2 | 2.0 | 0.41 |
|  |  |  |  |
| SOFA (points) | 18 | 20 | **0.005** |
| SAPS II (points) | 77 | 79 | 0.58 |
| MELD (points) | 33 | 27 | **0.0001** |

ALT, alanine transaminase; AST, aspartate transaminase; GGT, gamma-glutamyltransferase; L, liter; LDH, lactate dehydrogenase; MELD, Model for End-Stage Liver Disease; PTT, partial thromboplastin time; SAPS II, Simplified Acute Physiology Score II; SOFA, Sequential Organ Failure Assessment.

**Supplementary Table 3.** Comparison of relative changes in relevant biochemical variables and prognostic clinical scores after adjuvant extracorporeal liver support treatment for critically ill patients with hyperbilirubinemia and acute kidney injury caused by acute-on-chronic liver failure (ACLF). Patients were treated either with a combination of CytoSorb plus continuous veno-venous hemodiafiltration or with the ADVOS system. All values are given as percentages.

| **Median relative reduction (%)**  **Variables** | **CytoSorb n=31** | **ADVOS n=66** | **p value** |
| --- | --- | --- | --- |
| ALT | 5.5 | -37.3 | **0.04** |
| AST | -13.9 | -61.2 | **0.05** |
| GGT | 20.4 | 8.1 | 0.48 |
| LDH | -1.2 | -47.2 | **0.02** |
| Leukocytes | -16.1 | -11.7 | 0.30 |
| Hemoglobin | 9.9 | 4.0 | 0.19 |
| Platelets | 45.0 | 38.3 | 0.54 |
| Prothrombin time | 22.7 | 20.0 | 0.82 |
| PTT | -31.8 | -44.7 | 0.41 |
| Serum creatinine | 23.9 | 43.4 | **0.03** |
| Blood urea nitrogen | 23.5 | 56.1 | **0.01** |
| Lactate | -28.6 | -44.4 | 0.36 |
| pH | 0.2 | -0.6 | **0.05** |
| Base excess | 12.1 | 54.4 | 0.55 |
| C-reactive protein | -2.3 | 15.8 | 0.30 |
| Procalcitonin | 5.2 | 2.2 | **0.0001** |
|  |  |  |  |
| SOFA | 0.0 | -5.4 | **0.02** |
| SAPS II | 2.3 | -1.3 | 0.20 |
| MELD | 4.0 | 12.2 | 0.33 |

ADVOS, advanced organ support; ALT, alanine transaminase; AST, aspartate transaminase; GGT, gamma-glutamyltransferase; L, liter; LDH, lactate dehydrogenase; MELD, Model for End-Stage Liver Disease; PTT, partial thromboplastin time; SAPS II, Simplified Acute Physiology Score II; SOFA, Sequential Organ Failure Assessment.
